# Nonlinear mixed-effects model reveals a distinction between learning and performance in intensive reach training post-stroke

**DOI:** 10.1186/s12984-017-0233-2

**Published:** 2017-03-17

**Authors:** Hyeshin Park, Nicolas Schweighofer

**Affiliations:** Biokinesiology and Physical Therapy, University of Southern California, Los Angeles, CA USA

**Keywords:** Stroke rehabilitation, Reach training, Nonlinear statistical mixed-effects model, Learning, Performance, Activity-dependent fatigability, Prediction of long-term gains

## Abstract

**Background:**

We recently showed that individuals with chronic stroke who completed two sessions of intensive unassisted arm reach training exhibited improvements in movement times up to one month post-training. Here, we study whether changes in movement times during training can predict long-term changes.

**Methods:**

Sixteen participants with chronic stroke and ten non-disabled age-matched participants performed two sessions of reach training with 600 movements per session. Movement time data during training were fitted to a nonlinear mixed-effects model consisting of a decreasing exponential term to model improvements of performance due to learning and an increasing linear term to model worsening of performance due to activity-dependent fatigability and/or other factors unrelated to learning.

**Results:**

For non-disabled age-matched participants, movement times gradually decreased overall during training and overall changes in movement times during training predicted long-term changes. In contrast, for participants post-stroke, movement times often worsened near the end of training. As a result, overall changes in movement times during training did not predict long-term changes in movement times in the stroke group. However, improvements in movement times due to training, as estimated by the exponential term of the model, predicted long-term changes in movement times.

**Conclusion:**

Participants post-stroke showed a distinction between learning and performance in unassisted intensive arm reach training. Despite worsening of performance in later trials, extended training was beneficial for long-term gains.

## Background

Because of large differences in responsiveness to motor training post-stroke (e.g., [1]), predicting response to a specific training program is needed to improve outcomes via personalized treatment [2]. One possible approach is to observe the changes in motor performance during the early phase of training and adjust the training program accordingly, assuming that improvements in performance during training correlate with long-term improvements. However, because of activity-dependent fatigability – defined as a decline in strength during muscle groups’ use, fatigue, or reduced attention in long and repetitive training sessions [3, 4] – individuals post-stroke could show no improvements or even worsening of performance during training, but improvements in delayed retention tests. On the contrary, because of short-term components of motor memory post-stroke (e.g., [5]), motor performance could improve during training, but these improvements could be short-lived. Such learning-performance distinction has been extensively studied in motor learning (e.g., [6, 7]), but despite its clinical importance, it has been little studied in individuals with motor impairments due to stroke.

In this study, we re-analysed arm movement time during reach training in sixteen participants with chronic stroke collected from our recent study [8] and analysed new data in ten non-disabled age-matched participants. All participants performed two sessions of unassisted intensive training with 600 reaching movements per session to an array of five targets of fixed diameter. Our main objective was to determine whether changes in movement times during the first training session could predict changes in movement times between a pre-training test and a 1-month retention test in both participants post-stroke and non-disabled age-matched participants.

Our measure of performance was Movement Time (MT) for the following reasons. First, because we collected kinematic hand trajectory data, MT was available after every movement, allowing us to analyse a large number of repeated performance measurements. Second, we have recently shown [8] that two sessions of intensive arm reach training resulted in a significantly decrease in MT up to one month following training in individuals with chronic stroke. Third, a number of studies have used MT to quantify training-induced improvement in motor impairment and activity of the upper limb post-stroke [9–13]. Fourth, shorter movement times post-stroke are associated with better shoulder-elbow movement coordination during reach practice [11, 14–18]. Fifth, and most important, previous studies of reaching post-stroke [8, 19] showed that kinematic variables, including MT, can be linked to clinical scores such as the upper extremity Fugl-Meyer (FM). Note that here, we only used data from the first training session to predict long-term changes for two reasons. First, practically in the clinic, we want to determine if training will be effective as soon as possible for individual patients (to avoid “rehabilitation in vain”). Second, most of the changes in movement time due to training occurred in the first session (see Fig. 3C in Park et al. [8]).

We developed nonlinear mixed-effects models to decompose changes of MT during training into both gradual improvements of performance attributed to motor learning, as modelled with an exponential term that decreased as a function of training trials, and into gradual worsening of performance attributed to learning-unrelated factors [3], as modelled with a linear term that increased as a function of trials. The use of exponential term to model learning is motivated by the well-known negatively accelerated gains in performance as a function of training in most motor learning tasks [20], and by a recent study showing that long-term (one month) retention gain following arm reach training was predicted using a simple exponential decay model in non-disabled individuals [21]. The use of the linear term to model worsening in performance during training following an initial improvement is based on observing that many participants post-stroke complained about “being tired” during training. In addition, the experimenter (HP) noticed that the performance of several participants post-stroke appeared to worsen during training. Visual inspection of the MT data during training reinforced these observations, with several participants showing increases in MT during training. Although activity-dependent fatigability is a probable cause especially given our intensive training program, other factors such as fatigue, attention, and motivation, may influence performance during training [4, 22].

The use of the mixed-effects in the nonlinear model in this study is motivated by the high variability in lesion, impairment, spontaneous recovery, and responsiveness to therapy post-stroke [23, 24]. Such large variability in both initial performance and gains due to therapy was observed in our previous study [8]. One way to capture this variability is to fit a single model for each subject and then test for group differences for each parameter but such an approach poorly captures the underlying phenomena, because the large variability in parameters results in low power. Moreover, the risk of over-fitting the data is very high, with N*k parameters, where N is the number of participants and k the number of parameters to estimate. In contrast, an equivalent mixed-effects has only 2*k parameters: a mean (fixed parameters) and a variance (random parameters) around this mean for each or the k parameters. Thus, a single mixed-effects model can account for the large between-individual variability in initial and final performance, in learning-related performance changes, and in learning-unrelated performance changes. The individual parameters (the random effects) can be estimated based on the data and the parameter distribution [25]. Although such nonlinear mixed-effects models are commonly used to model repeated measures in pharmacokinetic analysis, for instance, to describe drug concentration in the bloodstream, [26], such models have only been recently used to characterize motor learning [27, 28].

We thus hypothesized that a nonlinear mixed-effects model with exponential and linear terms will: 1) capture the between-subject variability in initial performance, 2) capture the differences in rate of performance improvements with training, 3) capture worsening in performance during training that is presumably due to fatigue or activity-dependent fatigability [3, 4], and 4) predict the long-term (1 month) retention due to training. We also hypothesized that the participants with stroke will show larger worsening of performance during training than the non-disabled age-matched participants as shown by a larger “fatigue” model parameter. Finally, to predict the long-term effect of training, we studied the relationship between the model parameters and baseline initial movement time and baseline upper extremity Fugl-Meyer (FM) scores.

## Methods

### Participants

Twenty-six participants were included in this study: sixteen participants with chronic stroke with mild to moderate impairments (63.2 ± 2.7 years; 14 males, 2 females), referred to as the stroke group, and ten non-disabled age-matched participants (56.6 ± 2.9 years; 5 males, 5 females), referred to as the control group. This study presents a re-analysis of the data of the sixteen participants in the stroke group from our previous study [8]. The ten participants in the control group provided baseline data for the previous study, but received the same reach training and test paradigms as the sixteen participants post-stroke for the purpose of the present study. Potential participants in the stroke group were included in [8] if they were at least 6 months post-stroke, were able to follow and remember instruction (Mini-Mental State Examination score > 25/30) [29], scored upper extremity FM motor test > 19/66, and were able to reach to the farthest target of the Arm Reach Training system table within seconds. Exclusion criteria of participants with stroke were: 1) presence of any neurologic diagnoses other than stroke, 2) orthopedic disorders affecting the paretic upper extremity, 3) severe pain when moving the arm, and 4) visual neglect (more than 4% of lines left uncrossed on Albert’s test) [30]. Only right-handed individuals were included in the control group according to the Edinburgh Handedness Inventory [31] but excluded if they had any central neurological dysfunction. Detailed demographic information for all participants can be found in Table 1. The study was approved by the University of Southern California Institutional Review Board. All participants were naïve to the purpose of the experiments, and read and signed an informed consent prior to enrolment.

**Table 1:** Demographic information for the participants in the two groups

	Subject	Age (years)	Gender	MMSE (30 max)	Side affected or hand dominant side	Stroke duration (month)	Baseline FM (66 max)
Stroke group	S1	79	M	29	R	143	55
	S2	46	M	28	L	42	58
	S3	55	M	28	R	72	53
	S4	58	M	27	R	69	45
	S5	72	M	26	L	105	52
	S6	81	M	30	L	130	40
	S7	57	M	26	L	70	63
	S8	55	F	30	L	19	45
	S9	67	F	29	R	109	40
	S10	45	M	30	R	12	30
	S11	59	M	29	R	24	53
	S12	71	M	28	R	13	65
	S13	71	M	30	L	118	41
	S14	57	M	30	R	22	51
	S15	73	M	29	L	105	55
	S16	65	M	30	L	100	33
	mean ± SE	63.2 ± 2.7	14 M/2 F	28.7 ± 0.4	8R/8 L	72 ± 11	48.7 ± 2.5
Control group	S1	47	M	30	R		
	S2	53	M	27	R		
	S3	44	M	29	R		
	S4	69	M	29	R		
	S5	49	F	30	R		
	S6	53	M	28	R		
	S7	62	F	30	R		
	S8	55	F	30	R		
	S9	70	F	29	R		
	S10	63	F	30	R		
	mean ± SE	56.6 ± 2.9	5 M/5 F	29.2 ± 0.3	10R		

*MMSE* Mini-mental state examination scores, *FM* upper extremity score of Fugl-Meyer motor test, *SE* Standard error

### Study design, experimental setup, and timeline

The study design, experimental setup, and timeline for each trial are briefly described below; refer to Park et al. [8] for a complete description. All participants underwent two sessions of unassisted intensive reach training of 600 trials with trunk restraint and visuo-auditory feedback about movement speed. Results from our previous study [8] showed that participants post-stroke improved by more than 20% from pre-training to one month post-training on average in MT, movement smoothness, and a measure of upper extremity function (Box and Block test).

**Fig. 1:**
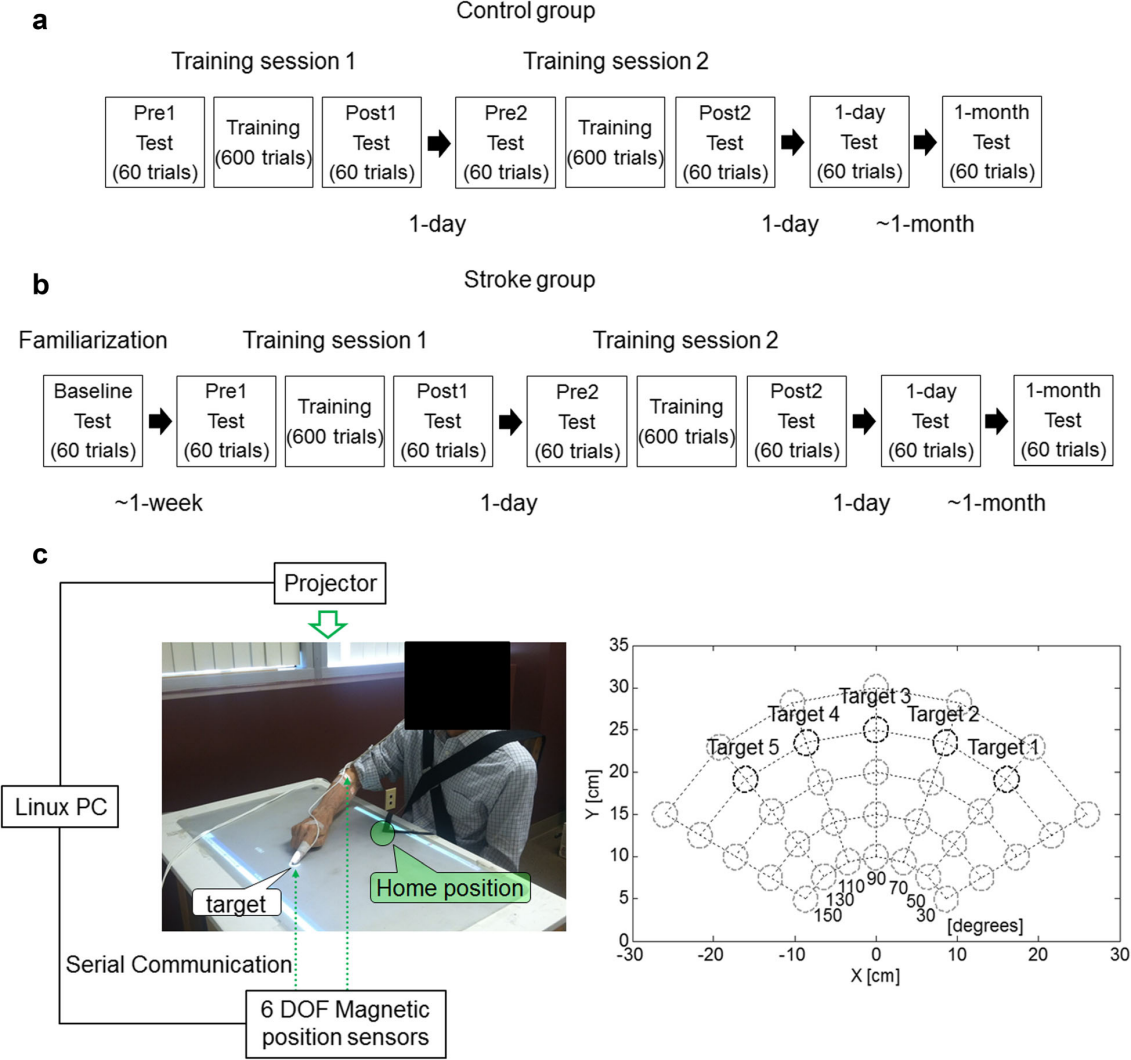
Experimental design and the Arm Reach Training system. **a** Diagram showing the timing of the four visits over a 5-week period for the control group. **b** Diagram showing the timing of the five visits over a 6-week period for the stroke group. **c** Left: Arm Reach Training system: the home-position is identified by the green circle and a target by the white circle. For each trial, participants were instructed to reach to the target with their index fingertip (of more affected hand in the stroke group and dominant hand in the control group) as quickly as possible. Right: Diagram showing the location of the 35 test targets in the two dimensional workspace. The five targets at 25 cm (black circles) are the training targets

During both test and training, participants were seated in a wood chair with a seatbelt restraining trunk movements in front of a wood table [18]. All participants visited the laboratory for four and five visits over a 5- and 6-week period, respectively. At the first visit, participants in the stroke group undertook clinical tests and a baseline test to familiarize themselves with the Arm Reach Training system (Fig. 1c). Participants in the stroke group within a week after the first visit and participants in the control group visited the lab for three days in a row for two training days, followed by a 1-day retention test. Participants in both groups visited the laboratory once more in the 4th week following training for a 1-month retention test (Fig. 1a and b). The two sessions of intensive training on consecutive days consisted of 600 movements split into six blocks, with 100 trials for each block, and with at least five-minute rest periods between blocks. Each movement was directed to one of five training targets (diameter 3 cm) projected onto the table and displayed in pseudo-random order. Participants performed a total of six arm reach tests of 60 movements each: Pre1 (before training) and Post1 (after training) tests in the first training day, Pre2 and Post2 tests in the second training day, and retention tests at 1-day and at 1-month post-training (Fig. 1a and b). In each test, participants made five reaching movements to each of the five training targets to test for training-induced generalization effect to non-trained targets, and then one reaching movement to each of 35 testing targets arrayed on an arc ranging from 30 to 150° and at 10, 15, 20, 25, and 30 cm (Fig. 1c).

At the beginning of each trial, all participants were instructed to place their index finger on a home-position (Fig. 1c, left panel). After a target appeared on the table and a “Go” sound was played, participants were instructed to move their arm to position the index finger on the target as quickly as possible [9]. After the target disappeared, participants were instructed to return the index finger to the home-position. After 1 s, the next target appeared in a pseudo-random order at one of the other four locations during training, or at one of the 34 non-training locations during testing. The maximum movement time allowed was 5 s. Following each training trial, one of five visuo-auditory feedback signals was played based on the participant’s deviation from the movement time mean and standard deviation in the preceding trials (see Park et al. [8] for details). To monitor reach performance during both training and testing, a first magnetic sensor was attached to the index fingertip and a second one to the lateral epicondyle of the humerus of the more affected hand (stroke group) and the dominant hand (control group) as shown in Fig. 1c.

### Data analysis

To estimate MT, the sensor position data were low-pass filtered with a cutoff frequency of 5 Hz using second-order Butterworth filter using MATLAB (The MathWorks, Natick, MA). Then, the velocity data were computed as the first derivative of the position data. MT was computed using the velocity data of the index fingertip. MT indicates the time interval between the instant when the fingertip’s velocity first exceeded 5% of maximum velocity [32] and the instant at which the fingertip’s velocity fell below 5% of maximum velocity when the fingertip was located inside the target. For participants with right hemiparesis, movement to leftward targets are slower and contain more peaks in the velocity profile than movements of the same extent but to the rightward targets; for participants with left hemiparesis, the opposite is true [8]. Because of the limited number of participants, MTs from all participants were analysed at once, by “flipping” the MT data from participants with left hemiparesis from the left to right, so that left hemiparetic participants behaved as right hemiparetic participants.

### Nonlinear statistical model with mixed-effects

MTs during the first training session were modelled with a nonlinear mixed-effects model consisting of a noise term and the sum of the following terms: a decreasing exponential term to model improvement in MT due to motor learning, an increasing linear term to model gradual worsening in MT due to possible fatigability and/or other factors unrelated to learning, and constant terms to model asymptotic MT for each of the five training targets. The model was fit based on the MT data of the 26 participants.

Specifically, the nonlinear model with mixed-effects was formulated as:

$$ M{T}_{i, j, k}={A}_i\times {e}^{-\frac{t_j}{ t a{ u}_i}}+{C}_i\times {t}_j+{D}_{i, k}+{\varepsilon}_{i, j, k} $$

where *MT*_*i,j,k*_ was the MT for participant number *i* (*i* = 1 ~ 26), trial number *t*_*j*_ (*j* = 1 ~ 120), and training target number *k* (*k* = 1 ~ 5), A_i_ was the mixed-effects exponential amplitude parameter, tau_i_ was the mixed-effects decay rate parameter, *C*_*i*_ was the mixed-effects “fatigue” parameter, *D*_*i,k*_ was the mixed-effects intercept term for target *k*, and ɛ_*i,j,k*_ was a noise term. *A*_*i*_ and *D*_*i,k*_ were expressed in milliseconds (ms) and tau_i_ was expressed in trials, whereas *C*_*i*_ was expressed in ms/trial. MTs were modelled for each of the five training targets separately via the *D*_*i,k*_ terms (with 120 trials for each training target), because MTs differed for targets at different angles. For instance, in non-disabled individuals, reaching movements with the right arm are slower to leftward than rightward targets [33], because leftward movements require coordinated movements from the shoulder and elbow and compensation for interaction torques [33, 34]. Similarly, in post-stroke individuals, leftward movements show the most deficits in control of limb dynamics [14] as well as larger MTs and lower smoothness before and after motor training [10, 16]. Comparisons of model fits using the root-mean-square error (RMSE) showed that additions of the target-dependent asymptotic term and fatigue term improved model fit.

**Fig. 2:**
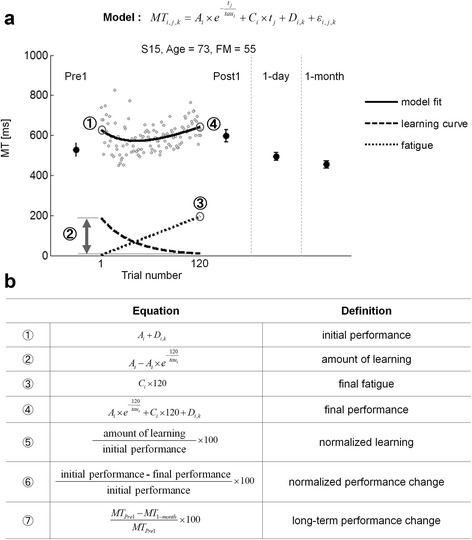
**a** An example of MT data and fitted model for Target 5 for subject 15 in the stroke group. Note how the model captures the initial decrease and the later increase in performance, as defined by MT. Gray dots represent individual trial-by-trial MT. Solid black lines represent the model fit; dashed lines represent the learning curve; dotted lines represent the estimated “fatigue”. MT_Pre1_ and MT_1-month_ represent mean MT in Pre1-test and in 1-month retention test, respectively. Pre1, Post1, 1-day, and 1-month show mean ± SE MT in Pre-test, immediate Post-test, 1-day retention, and 1-month retention test, respectively. Absolute quantities ➀ to ➃ in the table are illustrated in (**b**), which defines the performance and learning variables

Figure 2a illustrates the model fit to MT data during training for one participant post-stroke. Figure 2b defines the following important variables: *initial performance* corresponds to initial estimated MT; *amount of learning* is the improvement in MT during training estimated by the amplitude of the decreasing exponential between the beginning and the end of training; *normalized learning* is *amount of learning* normalized by *initial performance*; *final fatigue* is the worsening in performance estimated by the linear increase term at the end of training; *normalized performance change* is the change in performance between the beginning and the end of training predicted by the complete model normalized by *initial performance*; and *long-term performance change* is the difference in mean MTs in Pre1-test and 1-month retention test normalized by mean MT in Pre1-test.

Model parameters were estimated using the MATLAB *nlmefitsa* function that allows fitting a nonlinear mixed-effects regression model. To evaluate goodness of fit for each group, RMSE values obtained from the MATLAB *nlmefitsa* function for MT as a function of the trial number were expressed in ms. Possible correlation between the model parameters and baseline upper extremity FM scores as well as initial MT in Pre1-test were also tested.

Shapiro-Wilk normality test was used to check normality. Normally distributed data were expressed as mean ± standard error (SE) of the corresponding mean. Non-normally distributed data were expressed as median with interquartile ranges (IQR), 25% – 75%, and compared using the Mann–Whitney tests. Correlation for model parameters and baseline performance and impairment was examined with the Pearson test. The level of statistical significance was set at *p* < 0.05.

## Results

### Demographic information

For the stroke group, the average stroke duration was 72 ± 11 months and upper extremity FM score was 48.7 ± 2.5 (Table 1). There was no difference in age between the stroke and control groups (*t*-test, *p* = 0.117) [8]. All participants had a score of 0 on Albert’s test, that is, exhibited no visual neglect.

All participants completed the two training sessions (600 trials each session) by successfully reaching all targets within 5 s. Performing the 600 movements in the first training session, including break times, lasted on average 107.2 ± 4.6 min (range: 63.9 – 143.5 min) in the stroke group and 91.6 ± 5.9 min (range: 64.7 – 117.7 min) in the control group.

### Nonlinear statistical model with mixed effect in movement time

**Fig 3:**
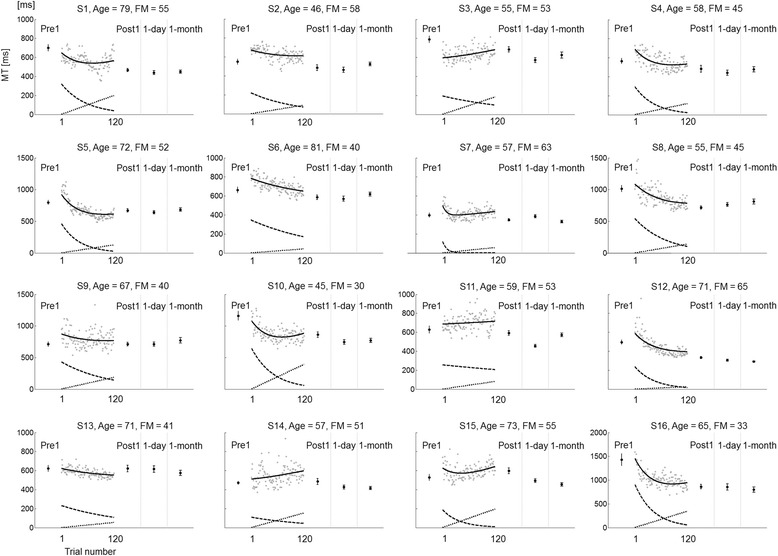
Individual MT for reaching movements to Target 5 and model fit in the stroke group (*n* = 16). Gray dots represent individual trial-by-trial MT during training. Solid black lines represent the model fit; dashed lines represent the learning curve; dotted lines represent the estimated “fatigue”. Pre1, Post1, 1-day, and 1-month show mean ± SE MT in Pre-test, immediate Post-test, 1-day retention, and 1-month retention test, respectively

**Fig. 4:**
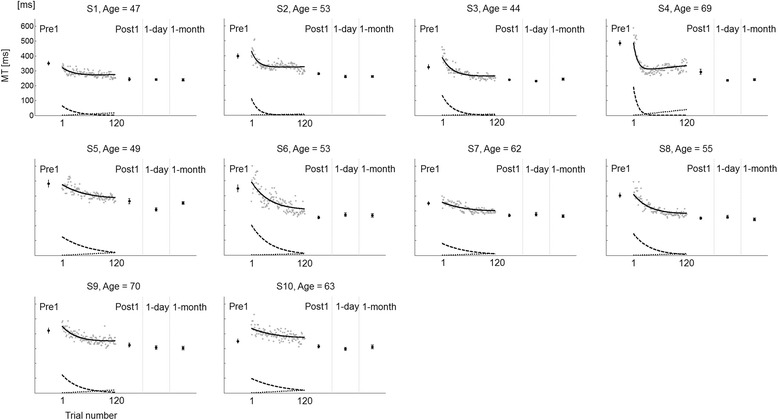
Individual MT for reaching movements to Target 5 in the control group (*n* = 10). Note the very small amount of “fatigue” compared to the stroke group. Data and model output as in Fig. 3

Figures 3 and 4 show MT data from Target 5 only (the right-most target located at 130° (0° rightward direction aligned on the torso); the changes in MT are qualitatively similar for the four other targets (plots not shown). Due to the mixed-effects parameters, the model provided a good fit to each participant’s MT data despite large individual differences in initial MT, changes in MT during training, and final MT. The nonlinear mixed-effects model provided overall good fit to each participant’s MT for reaching movements. RMSE on average was 24 ± 3 ms in the control group and 76 ± 4 ms in the stroke group. Thus, in the two groups, RMSE was about 10% of the mean MTs at the onset of training. Most participants exhibited initial improvements in MTs, except for subjects 3 and 14. Many participants, however, experienced a gradual worsening after an initial improvement in MT (see for example subjects 1, 7, 10, and 15). The worsening was modelled by a relatively large effect of “fatigue” for most participants (dotted upward straight lines below the data in Fig. 3). For most participants, performance in delayed retention tests appeared better than performance at the end of training (see below for additional analysis).

Figure 4 shows the MT data and model fit for the control group. All participants in the control group showed improvement in MT. Unlike the stroke group, all participants showed very little effect of “fatigue” (see below for statistics). Most showed performance in delayed retention test similar to performance at the end of training (see below).

**Fig. 5:**
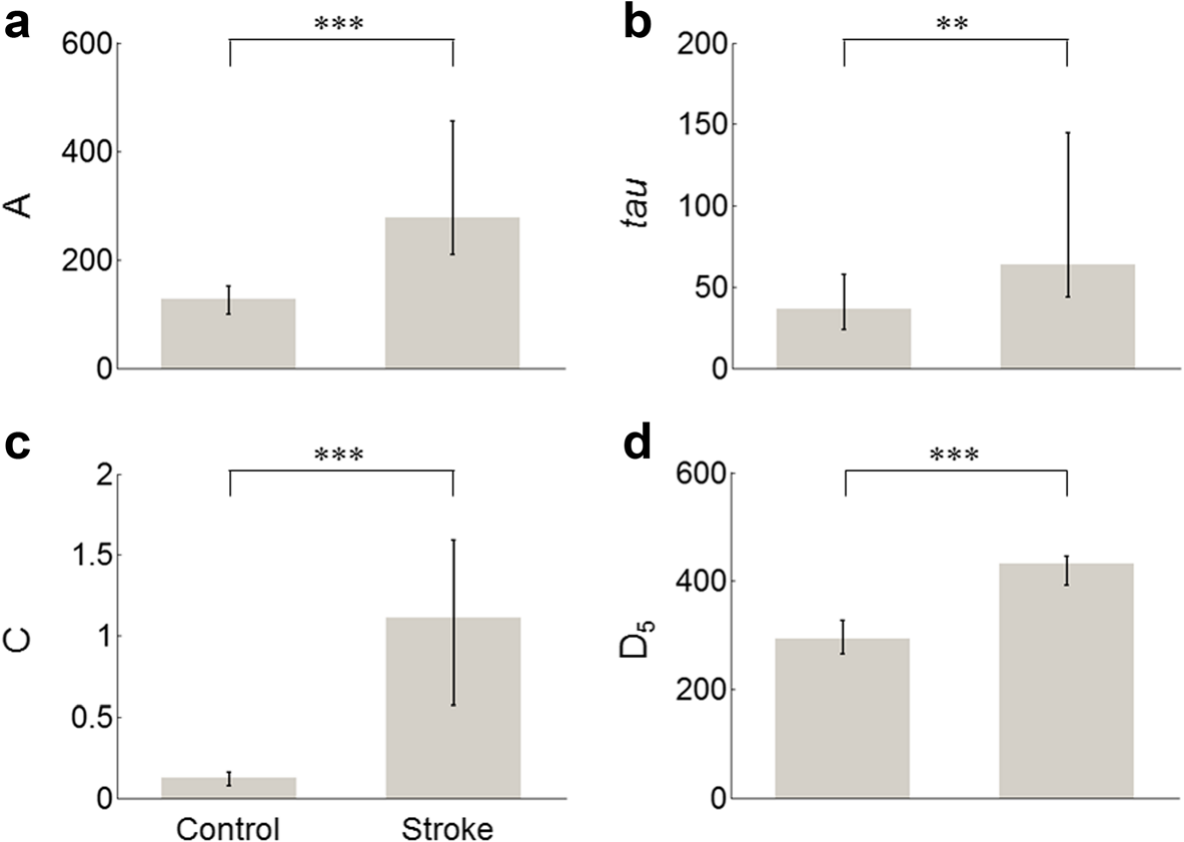
Model parameters of model for the control and stroke groups. Gray bars represent the median for *A*, *tau*, *C*, and *D*_*5*_. Error bars indicate the interquartile range 25% – 75% for *A*, *tau*, *C*, and *D*_*5*_ in each group. * *p* < 0.05, ** *p* ≤ 0.01, *** *p* ≤ 0.001

Figure 5 shows the median and interquartile range of the model parameters for the two groups. Nonparametric analyses were performed for all parameters, *A*, *tau*, *C*, *D*_*1*_, *D*_*2*_, *D*_*3*_, *D*_*4*_, and *D*_*5*_, which were not normally distributed based on the results of Shapiro-Wilk tests (all *p* < 0.0001). The parameters in the stroke group were significantly larger than the parameters in the control group (Fig. 5). The median exponential amplitude *A* was significantly larger in the stroke group (*A* = 279 ms, IQR 210 to 455 ms) than in the control group (*A* = 128 ms, IQR 100 to 151 ms, Mann–Whitney test *p =* 0.001) (Fig. 5a). The median decay parameter *tau* was significantly larger in the stroke group (*tau* = 64 trials, IQR 44 to 145 trials) than in the control group (*tau* = 36 trials, IQR 24 to 58 trials, Mann–Whitney test *p =* 0.01) (Fig. 5b). The median fatigue parameter *C* was more than nine times greater in the stroke group (*C* = 1.11, IQR 0.57 to 1.59) than in the control group (*C* = 0.12, IQR 0.07 to 0.16, Mann–Whitney test *p* < 0.0001) (Fig. 5c). The median asymptotic performance *D*_*1*_, to *D*_*5*_ monotonically increased from target 1 to 5 in the stroke group (from D_1_ = 328 ms, IQR 305–366 ms to D_5_ = 432 ms, IQR 392–445 ms) and in the control group (from D_1_ = 227 ms, IQR 212–269 ms to D_5_ = 293 ms, IQR 266–327 ms), with all *D*_*i,k*_ higher in the stroke group compared to the control group (all Mann–Whitney test *p* < 0.0001) (Fig. 5d).

### Prediction of long-term performance

**Fig. 6:**
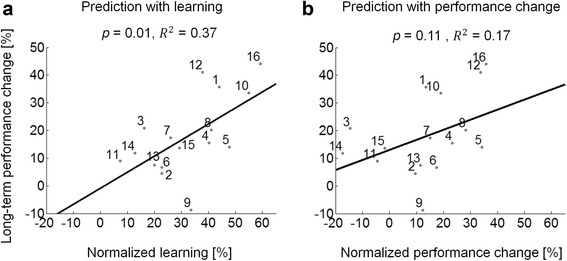
Predicting long-term changes from changes during training for the stroke group (*n* = 16). **a** Significant linear relationship between *normalized learning* estimated by the exponential term in the model and *long-term performance change*. **b** Non-significant linear relationship between *normalized performance change* and *long-term performance change*. Solid black lines represent least-squares fit to individual data in the stroke group

In the stroke group, *normalized learning* showed a significant linear relationship with the change in MT between pre-test and 1-month post-test, i.e., *long-term performance change* (*R* ^2^ = 0.37, *p* = 0.01; without subject nine who showed no improvement in long-term performance: *R* ^2^ = 0.51, *p* = 0.003) (see Fig. 6a). There was no significant linear relationship between *normalized performance change* and *long-term performance change* (*R* ^2^ = 0.17, *p* = 0.11; without S9: *R* ^2^ = 0.22, *p* = 0.08) (see Fig. 6b). Similarly, the control group showed a significant linear relationship between *normalized learning* and *long-term performance change* (*R* ^2^ = 0.61, *p* = 0.008). Unlike the stroke group, there was a significant linear relationship between *normalized performance change* and *long-term performance change* (*R* ^2^ = 0.51, *p* = 0.02). This latter result was expected because the effect of “fatigue” was near zero (median *C* parameter, Fig. 5c and above) in these participants; therefore, there was a small difference between *normalized learning* and *normalized performance change.*

### Relationship between individual model parameters and baseline performance and impairment

In the stroke group, the initial upper extremity FM scores showed a significant negative correlation with the mixed-effects amplitude parameters *A* (*r* = −0.725 and *p* = 0.002), the fatigue parameter *C* (*r* = −0.619 and *p* = 0.011), and with asymptotic performance *D*_*5*_ (*r* = −0.511 and *p* = 0.043), but not with the decay parameter *tau* (*r* = 0.021 and *p* = 0.940) (Table 2). In addition, MT_Pre1_ showed highly significant correlations with *A* (*r* = 0.945 and *p* < 0.0001), *C* (*r* = 0.774 and *p* = 0.0004), and *D*_*5*_ (*r* = 0.686 and *p* = 0.003), but not with *tau* (*r* = −0.129 and *p* = 0.635) (Table 2). In the control group, MT_Pre1_ showed significant correlations with *A* (*r* = 0.681 and *p* = 0.03), but not with *tau* (*r* = −0.114 and *p* = 0.755), *C* (*r* = 0.489 and *p* = 0.151), or *D*_*5*_ (*r* = 0.475 and *p* = 0.166) (Table 2).

**Table 2:** Correlation coefficient (*r*) between mean model parameters of each group and MT_Pre1_ and between mean model parameters and upper extremity FM scores in the stroke group

		A	tau	C	*D*_*5*_
Control group	MT_Pre1_	0.681^*^	−0.114	0.489	0.475
Stroke group	MT_Pre1_	0.945^***^	−0.129	0.774^***^	0.686^**^
	FM	−0.725^**^	0.021	−0.619^*^	−0.511^*^

Pearson correlation for *A*, *tau*, *C*, and *D*_*5*_ in the two groups. * *p* < 0.05, ** *p* ≤ 0.01, *** *p* ≤ 0.001

## Discussion

We proposed a novel nonlinear statistical model with mixed-effects that accounted for the immediate and delayed changes in performance due to intensive arm reach training in individuals with chronic stroke and non-disabled age-matched individuals. Performance was operationalized with MT, a performance variable available at each trial in our arm reach protocol. The model estimated improvement in MT due to motor learning via a decreasing exponential term, worsening of performance due to learning-unrelated factors via an increasing linear term, and asymptotic performance via target-dependent constant terms. Thanks to the mixed-effects, this single model, with only eight free fixed parameters, fit all data from post-stroke and non-disabled 26 participants simultaneously (see Figs. 3 and 4). The nonlinear mixed-effects model can be used in a variety of learning studies such as those with large variability across individuals, for instance [35].

Notably, we found that the learning-related exponential term predicted long-term changes in MT in the stroke group, but not by the overall change in performance during training (Fig. 6). Therefore, the model containing both negatively accelerated improvement and linear worsening of performance during training, “unmasked” a learning-performance distinction in intensive reach training post-stroke. Although fatigability, defined as a decline in strength during muscle groups’ use, is a probable cause for the worsening effect in the stroke group, especially given our intensive training program, increase in fatigue, decrease in attention, motivation, and other factors may affect performance during training [3, 4, 22]. In contrast, the control group showed no distinction between learning and performance, because the “fatigue” parameter was near zero.

All model parameters, i.e., the initial performance parameter *A*, the decay rate parameter *tau*, the “fatigue” parameter *C*, and the asymptotic performance terms *D*_*k*_, were significantly larger in the stroke group than in the control group. In contrast, the stroke group learned to decrease MT at a slower rate than the control group. The median half-life time of the exponential was 44 trials out of 120 trials for the stroke group versus 25 trials for the control group. As observed in Figs. 3 and 4, whereas the exponential term was still decreasing toward the end of the training for participants in the stroke group, the effect of training plateaued for the control group. This result shows that participants in the stroke group continued to benefit from motor practice even though “raw” performance often plateaus as early as mid-training (see for instance S1, S10, and S15 in Fig. 3).

A number of model parameters correlated with clinical measures. For instance, initial performance, asymptotic performance, and “fatigue” term in the stroke group were predicted by the baseline upper extremity FM scores and MT_Pre1_. In contrast, the decay parameter *tau*, which provides an estimate of the rate of motor learning, did not correlate with baseline performance or impairment levels, despite being larger in the control group. The rate of learning may correlate with measures of brain injury or cortical function. For instance, a recent study showed that smaller corticospinal tract injury and cortical greater ipsilesional motor cortex (M1) activation were better predictors of response to a robotic training program than baseline impairment predictors [1]. In addition, psychological variables such as self-efficacy may be important predictors in the rate of improvements in motor learning [36]. Finally, the integrity of short-term memory post-stroke may be useful in predicting both the rate of learning and final performance [5]. Using the same dataset, we previously reported that the MT_Pre1_ significantly correlated with the initial upper extremity FM scores (R^2^ = 0.53, *p* < 0.001) and that changes in the Box and Block tests between Pre1- and 1 month retention test significantly correlated with the changes in MT (R^2^ = 0.56, *p* = 0.001) [8]. Another kinematic variable, the number of peaks in the velocity profile, is an indicator of movement smoothness and positively relates with stroke recovery [8, 12, 37]. In our previous study, we found that MT_Pre1_ significantly correlated with the number of peaks at Pre1-test (R^2^ = 0.65, *p* < 0.002) [8]. In the present study, however, the initial number of peaks was small at the onset of training, especially in the control group, making the number of peaks unsuitable for fitting with a continuous regressor, such as exponential decay as a function of trials.

Because the present study was a retrospective analysis of existing data, the main limitation is that we did not measure factors that may account for the worsening in performance during training. Thus, it is not clear what the “fatigue” term in our proposed model represents. Although fatigability is a probable cause, especially given our intensive training program, fatigue, attention, motivation, and other factors may affect performance during training [3, 4, 22]. Future studies should repeatedly measure fatigability with strength testing and fatigue with visual analog scales before, during, and after training. A second limitation is that our proposed model may or may not be generalized to the wider stroke population since the participants in this study were in chronic stage (i.e., the minimum duration post-stroke since stroke 12 months) and with mild to moderate impairments. A future larger study should include different chronic stage and/or impairment level. A third limitation is that the accuracy requirements were fixed: all participants (stroke and control groups) were instructed to reach the targets (disk of 3 cm in diameter) as quickly as possible. Reaching anywhere within the target within the 5 s time limit was sufficient to successfully terminate the trial: all participants completed all trials within the 5 s time limit. Thus, accuracy is a fixed value; the effect of training on speed accuracy trade-off will require future work.

## Conclusion

We propose a new analysis of motor performance during the first session of motor training that can help clinicians decide whether such training is effective in improving arm and hand function in the long-term. In addition, estimates of motor learning during training (as operationalized by *normalized learning* in Figs. 2 and 6) could help clinicians to adjust the duration and/or the number of training sessions and determine the dosage of training for individuals post-stroke. Note however, that our proposed model predicted long-term changes, it only explained 37% (51% excluding a participant who did not improve out of 16 participants) of the variance. Models combining behavioral “learning” co-variates with neural co-variates [38, 39] could further improve predictions of the response to motor training post-stroke. The availability of such accurate models could transform neuro-rehabilitation programs because clinicians, patients, and insurance companies would be able to determine effective treatments while reducing costs.

## Abbreviations

FM: Upper extremity score of Fugl-Meyer motor test
IQR: Interquartile ranges
ms: Milliseconds
MT: Movement time
RMSE: Root mean square error
SE: Standard error
